# A Low-Cost Fiducial Reference Phantom for Computed Tomography

**DOI:** 10.6028/jres.113.027

**Published:** 2008-12-01

**Authors:** Zachary H. Levine, Steven Grantham, Daniel S. Sawyer, Anthony P. Reeves, David F. Yankelevitz

**Affiliations:** Photon Physics Group National Institute of Standards and Technology, Gaithersburg, MD 20899-8410; Large-Scale Coordinate Metrology Group National Institute of Standards and Technology, Gaithersburg, MD 20899-8211; Department of Electrical and Computer Engineering Cornell University, Ithaca, New York 14853; Weill Medical College Cornell University, New York, New York 10065

**Keywords:** centroid, Coordinate Measuring Machine, dimensional reference, medical phantom, second moment tensor, tomography

## Abstract

To detect the growth in lesions, it is necessary to ensure that the apparent changes in size are above the noise floor of the system. By introducing a fiducial reference, it may be possible to detect smaller changes in lesion size more reliably. We suspend three precision spheres with a precision structure built from pieces from a popular children’s building toy. We measure the distances between the centroids of the structures three ways; namely, with a high-precision mechanical method, micro computerized tomography, and medical computerized tomography. The three methods are in agreement, and also agree with the design values for the structure. It is also possible to pick a threshold so that the three spheres have their nominal volumes in the medical computerized tomography images. The use of volumetric measures allows the determination of lengths to much less than the voxel size using materials which have x-ray properties within the range of the human body. A suitable structure may be built with a very small parts cost.

## 1. Introduction

Computerized tomography (CT) in medicine is the method of choice to detect and measure change in cancerous lesions. A typical protocol is to have a patient examined by CT and then re-examined, perhaps, six months later. The radiologist is then faced with the problem of determining whether, and by how much, a given nodule has changed in size. A common measure of size is the Response Evaluation Criteria in Solid Tumors (RECIST), wherein the longest dimension of the lesion is recorded. A two-dimensional metric due to the World Health Organization (WHO) is frequently used as well [1]. Three-dimensional volumetric protocols are just emerging into use by the community [2].

A systematic error may occur if the image has changed size due to some change in the measuring instrument. While the shot noise of photon statistics represents an ultimate limit, in the “real world,” a radiologist may be faced with data taken using equipment which is different due to hardware or software upgrade, due to the use of an alternate machine possibly at a different hospital or clinic, or even due to the use of different operator-specific protocols. This may cause an apparent change in the size of a lesion, leading to a false positive diagnosis of cancer, or mask a true change in size, leading to a false negative diagnosis.

Here, we propose the use of an inexpensive fiducial reference phantom to help sort out these issues. We show that the fiducial reference is able to provide an accurate length standard in a medical CT. This may lead to a more accurate understanding of the sizes of lesions, leading to more accurate or earlier diagnosis, as well as less need to redo studies due to changes in equipment. We also make a speculative suggestion of a method for setting the threshold used to define the contour of a lesion.

## 2. Materials and Methods

We propose to make the phantom from three precision spheres, separated using a sandwich of two precision spacers. As we shall see, the centroids of the spheres may be determined to subvoxel accuracy in a medical CT. In choosing the material for the spheres, we note that any practical algorithm must allow for the reconstruction of materials with an absorption coefficient from air to bone. Using the scale of Hounsfield Units [3], this is a value between −1000 HU and +1000 HU. The Hounsfield Unit depends on the parameters of the CT scanner, e.g., the value of the high voltage and the target material which jointly determine the x-ray spectrum. (The HU does not appear in the *Système Internationale* (SI). However, it is necessary to report certain values in this paper because of their common use by the radiological community, and because the medical CT reports values in HU. These values are not easily convertible to SI.) For estimation purposes, we approximate the spectrum by a 70 keV mono-chromatic beam. Then, using the reference data [4], it is possible to estimate the absorption of various materials. These are shown in Table 1.

**Fig. 2:**
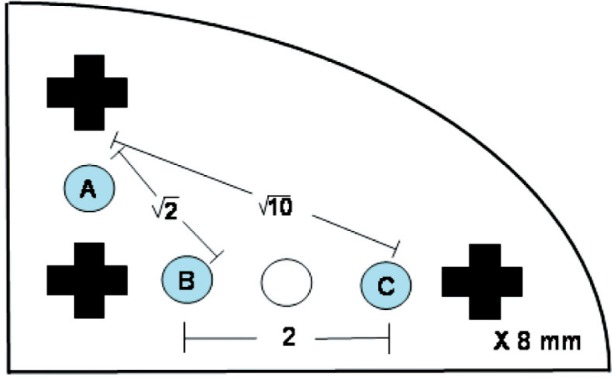
A schematic drawing of the LEGO 3 × 5 liftarm. The balls are placed at the locations marked A, B, C. The crosses represent holes for the crossaxles. All features are arranged on a squarae grid with 8 mm spacing.

We would like the fiducial reference to be widely deployed. To that end, we are interested in the construction of a low cost unit. We built the spacers out of LEGO^1^ pieces. In particular, we used two 3 × 5 liftarms in the structure along with three crossaxles and three bushings [5]. See Fig. 2 for a schematic drawing. The 3 × 5 liftarm allows for the insertion of 3 or 4 balls on a grid. We use 3 balls to ensure these are held securely. Based on our measurements of the liftarm, we determined that a 5.5 mm to 6.5 mm diameter ball would sit on the inner step of the stepped hole of the liftarm. (We measured the inner diameter of the stepped hole of the liftarm to be 4.86 mm and the outer diameter to be 6.14 mm. The thickness of the liftarm was found to be 3.88 mm and the step height was 0.81 mm.) We used a pair of liftarms to supply a symmetric support to each ball. We reasoned that any deformations induced by the support structure would lead to a symmetrical distortion of the balls and would not move their centroids. The liftarms are held in place by three crossaxles which have one open end and one tabbed end. Each cross axle was secured by a bushing. We estimate the cost of each LEGO piece to be US $0.10, or a total cost of US $0.80 for the structure. The balls are separately priced. We obtained polytetrafluoroethylene (PTFE, or Teflon) balls with nominal diameters of 6.35 mm (0.250 In) and specified as Grade 1, i.e., diameter and sphericity tolerance of ± 0.025 mm (± 0.001 In). (Sphericity is the greatest radial distance in any plane between a sphere circumscribed around the sphere and any point on the sphere.) In quantity, these also cost US $0.16 each, leading to a total parts cost of US $1.28. Hence, the sample is referred to as “low cost.”

We also report on the use of 6.0 mm diameter spherical BK7 glass lenses which were considerably more expensive and had an x-ray absorption value well in excess of that of bone, which is typically +1000 HU. However, as shown below, these had the virtue of being physically harder and therefore it was possible to determine their locations more precisely.

Our methods for determining the spacing between the balls are as follows. (1) LEGO pieces define an orthogonal grid with a spacing of 8 mm [6]. From Fig. 2, the balls may be taken to be at grid positions (0,1), (1,0), and (3,0) where the origin is at the center of the lower left crossaxle. Using the Pythagorean Theorem, the ball spacings should be $\sqrt{2}$, 2, and $\sqrt{10}$ times the 8 mm grid spacing, i.e., 11.314 mm, 16.000 mm, and 25.298 mm, respectively. (2) We used a Cordax RS-5 Coordinate Measuring Machine (CMM) at the National Institute of Standards and Technology to determine the positions of the balls in the structure. This particular CMM uses contact probe technology. (3) We obtained a microCT reconstruction with a voxel size of 0.028 mm, which is far below a typical medical CT voxel (about 0.5 mm). (4) We obtained a medical CT reconstruction using a GE Lightspeed.

The spheres were measured using the CMM with 15 points distributed over the exposed portion of the sample. The diameter was calculated as a least squares fit to the 15 measured points. Thermal corrections are negligible to the precision quoted. Traceability of the CMM scales was established by comparing the measured values of the sphere center coordinates to the measured values of a reference artifact that is traceable to the SI unit of length and positioned in the same nominal location on the CMM table.

For both the microCT and the medical CT, we obtained the centroid using the following procedure: first, a threshold value was chosen by hand to provide a good separation between the spheres and the support structure. In both cases, the PTFE balls had an x-ray absorption that was well separated from that of the liftarms. The central position was estimated from a representative slice near the ball center by hand using a mouse. A spherical mask with 150 % of the nominal radius was chosen; the value of 150 % was large enough to ensure that the whole sphere was included after the center was selected by hand but small enough so that no other sphere would be included. The object was taken to include all voxels above the threshold (below in the case of the microCT). The centroid of these voxels was determined. Additionally, using the same mask, the tensor of second moments about the centroid was found for each ball. That is, for each voxel above the threshold, a discrete sum was made of the product of two (not necessarily distinct) coordinate differences from the voxel center to the centroid determined above. Differences between these centroid positions and the second moment tensor are discussed in the Results section.

## 3. Results

The three measurements and the ideal values are compared in Table 2. The liftarms are made of acrylonitrile butadiene styrene (ABS) which has a measured peak absorption of + 18 HU, which is well separated from the PTFE balls with a measured peak absorption of + 905 HU or the glass lenses with a measured peak absorption of + 2684 HU.

The distances between the centroids of the balls on each sample are given in Table 2. The results represent subvoxel accuracy for medical CT. Our medical CT scans were taken with a voxel size of 0.703 mm × 0.703 mm × 1.250 mm. Medical CT voxel sizes are rarely less than 0.3 mm × 0.3 mm × 0.625 mm. The proposed fiducial reference has no dimension out of the ideal by as much as 0.1 mm. The data are drawn from a single scan for both medical CT and microCT.

We consider also the sizes of the individual spheres. The sphere diameters were measured with the CMM were 6.008 mm, 6.018 mm, and 6.048 mm, with a 95 % level of confidence uncertainty of ± 0.060 mm for the three glass spheres, compared to a manufacturer’s specification of 6.0 mm. For the three PTFE spheres, the results were 6.368 mm, 6.367 mm, and 6.401 mm, with an uncertainty of ± 0.095 mm, compared to a manufacturer’s specification of 6.350 mm ± 0.025 mm (quoted as 0.250 In ± 0.001 In). Hence all parameters measured are consistent with their specified or expected values given the uncertainties of measurement and the specified tolerances.

As discussed above, the sphere diameters were determined from the second moment tensor of the spheres after a threshold was applied to determine the pixels in each sphere. Theoretically, the second moment of a sphere of radius *R* is given by

$$\langle r^{2}\rangle =\frac{4\pi \int_{0}^{R}drr^{2}r^{2}}{4\pi \int_{0}^{R}drr^{2}}=\frac{3}{5}R^{2}.$$

Since 〈*r*^2^〉 = 〈*x*^2^〉 + 〈*y*^2^〉 + 〈*z*^2^〉, by symmetry 〈*x*^2^〉 = 〈*y*^2^〉 = 〈*z*^2^〉 = *R*^2^/5. Again, by symmetry, the off-diagonal elements of the second moment tensor vanish, i.e., 〈*xy*〉 = 〈*yz*〉 = 〈*zx*〉 = 0. The determinant *D* of the second-moment tensor for a sphere is therefore given by *D* = *R*^6^/125. The radii reported in Figs. 4 and 5 were obtained by finding the second moment tensor, then its determinant *D*, then applying *R* = (125*D*)^1/6^. We elected to use the determinant of the second moment tensor because it is a measure of the radius which takes into account most of the available data and is therefore expected to be more accurate than, for example, algorithms which rely on determining edges of the object.

**Fig. 3:**
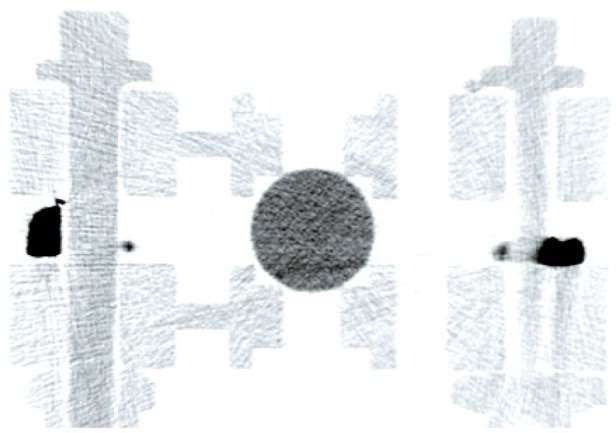
One slice from microCT reconstruction of the structure shown in Fig. 1. The microCT was acquired on a SkyScan 1172 by Micro Photonics, Inc. Ball A is shown in cross section, along with the two crossaxles which are located at the 10 cm mark in Fig. 1. The ball is seen to be in contact only with the inner ring. The dark structures at the sides were used to hold the figure in the microCT. Note the excellent contrast of the PTFE sphere in the center and the LEGO pieces.

**Fig. 4:**
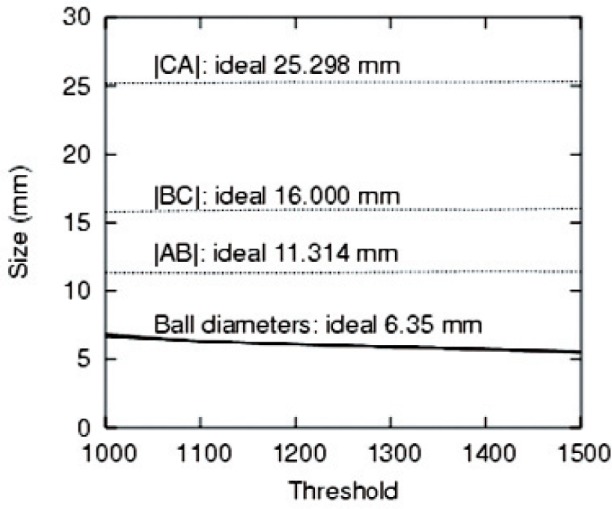
Distances between coordinates and sphere diameters as a function of the threshold (in arbitrary units).

**Fig. 5:**
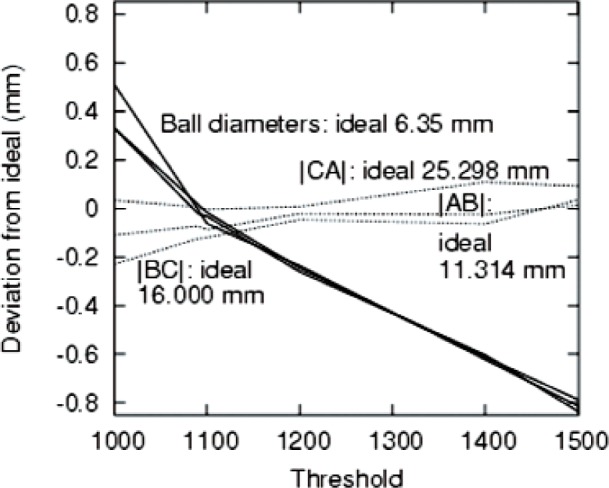
Deviations of the values plotted in Fig. 4 from their ideal values.

The components of the second moment tensor are given for the three PTFE balls for both medical CT and microCT. The values are sensitive to the threshold chosen. The values for Table 3 were chosen by a subjectively reasonable criterion to achieve good object segmentation with a single threshold value throughout the object. Several points may be noted. First, the microCT volumes are closer to the ideal values than the medical CT values. However, as shown in Fig. 3, by adjusting the threshold, many values, including the ideal value may be obtained within medical CT. Second, the off-diagonal components of the tensor are negligible. Third in two of three cases for the microCT, a prolate spheroid is observed; the other case is spherical. The 〈*z*^2^〉 values are fairly accurate for all three spheres in the case of microCT. For the medical CT, the spheres appear to be oblate although the lack of isotropy in the *xy* plane suggests that the more accurate statement is simply that the 〈*z*^2^〉 component is the smallest. It is difficult to draw conclusions from the limited number of conditions considered here. However, the distances identified as the difference between the centroids of the spheres are both more precise (due to the insensitivity to the threshold) and more accurate (closer to the ideal value) than distances based on an attempt to understand the spatial extent of the sphere in the reconstruction.

## 4. Discussion

A promising candidate for a fiducial reference phantom has been demonstrated. It offers low cost and simple assembly, sufficient precision for medical CT, and compatibility with the range of x-ray absorption found in the human body. The latter point implies that standard reconstruction algorithms will most likely give satisfactory reconstructions.

We suggest that the phantom may be useful in determining the sizes of lesions which have been taken under varying conditions. We further suggest that by tuning the threshold value to obtain a known volume, it may be possible to normalize lesions volumes. Since the difference in volumes is critical to the diagnosis of cancer, it may be possible to use smaller differences in volume to make more reliable or faster diagnoses.

## Figures and Tables

**Fig. 1:**
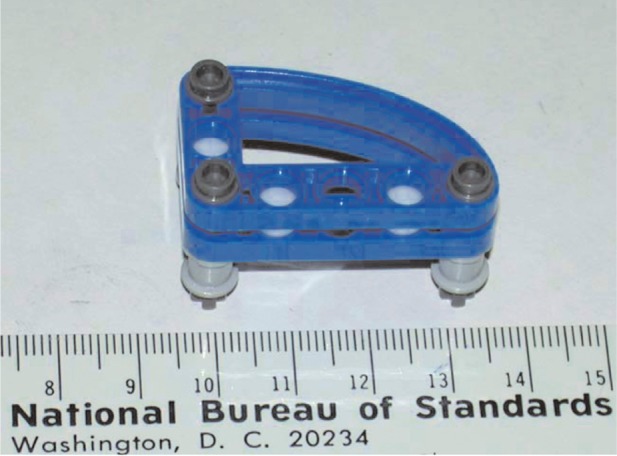
A photograph of the assembled LEGO structure shown with PTFE speres. The ruler is marked in cm.

**Table 1:** Absorption coefficients for 70 keV x rays and estimate of absorption of materials in HU. ABS is acrylonitrile butadiene styrene copolymer, and is reported to be the principle material in LEGO pieces. The attenuation coefficient of air is neglected in the estimate of the Hounsfield Units. The experiment is a maximum value taken reported by the medical CT. Air is −1000 HU and bone is typically + 1000 HU

Material	Attenuation coeff.^3^ (cm^2^/g)	Density Attenuation (g/cm^3^)	(cm) length	Est. HU	Exp. HU
Water	0.193	1.00	5.18	0 (by def.)	
ABS	0.180	1.05	5.29	−20	+18
PTFE	0.173	2.2	2.63	970	+905
Fused silica	0.195	2.2	2.33	1331	+2684

**Table 2:** Distances in the LEGO structure in mm. Here, points A, B, and C refer to the centroids of the balls indicated in Fig. 2. The ideal structure is discussed in the text. The method and material of the balls are given in each case. “Physical” refers to measurements performed at NIST on a coordinate measuring machine. MicroCT refers to data acquired by a SkyScan 1172 by Micro Photonics, Inc. with 0.028 mm voxels, and analyzed at NIST. Medical CT refers to data acquired by a GE Lightspeed at the Weill Medical College and analyzed at NIST. For distances other than the ideal, the deviations from the ideal values are listed. Uncertainties for the physical measurement have a coverage factor *k* = 2, which corresponds to a 95 % level of confidence. There is not sufficient experience to give uncertainties for the CT measurements

	|AB| (mm)	|BC| (mm)	|CA| (mm)
Ideal	11.314	16.000	25.298
Physical — PTFE	+ 0.013 ± 0.085	−0.039 ± 0.085	−0.039 ± 0.085
MicroCT — PTFE (deviation)	+ 0.032	+ 0.032	+ 0.050
Medical CT — PTFE (deviation)	+ 0.093	+ 0.036	+ 0.014
Physical — Glass (deviation)	+ 0.032 ± 0.055	−0.015 ± 0.055	+ 0.007 ± 0.055
Medical CT — Glass (deviation)	+ 0.025	+ 0.012	+ 0.061

**Table 3:** Components of the second moment tensor of the PTFE spheres in mm^2^. Voxel values were converted to mm using the nominal values of the machine settings, namely 0.703 mm × 0.703 mm × 1.250 mm for the medical CT and 0.028 mm for each dimension for the microCT. The values shown are for a threshold of 1500 on a scale of 0 to 4095 for the medical CT and 165 on an inverted scale of 255 to 0 for the microCT. The threshold values were selected to obtain a visually reasonable segmentation between the sphere and the supporting structure. The ideal value for the first three columns was found using the formula *R*^2^/5 with *R* = 3.175 mm

	〈*x*^2^〉	〈*y*^2^〉	〈*z*^2^〉	〈*yz*〉	〈*zx*〉	〈*xy*〉
Ideal	2.016	2.016	2.016	0.000	0.000	0.000
Medical CT A	1.546	1.592	1.464	0.034	−0.042	0.002
Medical CT B	1.608	1.535	1.502	0.025	−0.040	−0.028
Medical CT C	1.516	1.541	1.505	0.031	0.014	0.028
MicroCT A	1.918	1.930	1.928	−0.013	0.017	−0.012
MicroCT B	1.788	1.788	1.981	0.006	0.013	0.007
MicroCT C	1.791	1.795	1.970	−0.004	0.008	0.004

## Footnotes

1 Certain commercial equipment, instruments, or materials are identified in this paper to foster understanding. Such identification does not imply recommendation or endorsement by the National Institute of Standards and Technology, nor does it imply that the materials or equipment identified are necessarily the best available for the purpose.
